# Evaluation of Elecsys Syphilis Assay for Routine and Blood Screening and Detection of Early Infection

**DOI:** 10.1128/JCM.02544-15

**Published:** 2016-08-24

**Authors:** J. Kremastinou, V. Polymerou, D. Lavranos, A. Aranda Arrufat, J. Harwood, M. J. Martínez Lorenzo, K. P. Ng, L. Queiros, I. Vereb, M. Cusini

**Affiliations:** a Biomedicine SA, Athens, Greece; b Banco de Sangre y Tejidos de Aragon, Zaragoza, Spain; c Freeman Hospital, Newcastle, United Kingdom; d University of Malaya Medical Center, Kuala Lumpur, Malaysia; e Blood and Transplantation Center, Porto, Portugal; f Regional Hospital, Gävle, Sweden; g Fondazione IRCCS Ca' Granda Ospedale Maggiore Policlinico, Milan, Italy; Memorial Sloan-Kettering Cancer Center

## Abstract

Treponema pallidum infections can have severe complications if not diagnosed and treated at an early stage. Screening and diagnosis of syphilis require assays with high specificity and sensitivity. The Elecsys Syphilis assay is an automated treponemal immunoassay for the detection of antibodies against T. pallidum. The performance of this assay was investigated previously in a multicenter study. The current study expands on that evaluation in a variety of diagnostic settings and patient populations, at seven independent laboratories. The samples included routine diagnostic samples, blood donation samples, samples from patients with confirmed HIV infections, samples from living organ or bone marrow donors, and banked samples, including samples previously confirmed as syphilis positive. This study also investigated the seroconversion sensitivity of the assay. With a total of 1,965 syphilis-negative routine diagnostic samples and 5,792 syphilis-negative samples collected from blood donations, the Elecsys Syphilis assay had specificity values of 99.85% and 99.86%, respectively. With 333 samples previously identified as syphilis positive, the sensitivity was 100% regardless of disease stage. The assay also showed 100% sensitivity and specificity with samples from 69 patients coinfected with HIV. The Elecsys Syphilis assay detected infection in the same bleed or earlier, compared with comparator assays, in a set of sequential samples from a patient with primary syphilis. In archived serial blood samples collected from 14 patients with direct diagnoses of primary syphilis, the Elecsys Syphilis assay detected T. pallidum antibodies for 3 patients for whom antibodies were not detected with the Architect Syphilis TP assay, indicating a trend for earlier detection of infection, which may have the potential to shorten the time between infection and reactive screening test results.

## INTRODUCTION

Syphilis is a curable sexually transmitted infection (STI) caused by the spirochete Treponema pallidum subsp. pallidum (1). It can also be transmitted from mother to child *in utero* or during birth or, rarely, by transfusion of blood, blood components, or organs from donors with active syphilis (1–5). The disease can be divided into stages, based on clinical findings. The primary and secondary stages of the disease are characterized by initial skin manifestations, such as painless sores and macules, and symptoms such as tiredness and headaches, which may be mistaken for other conditions. If undetected, syphilis enters a lengthy latent period, defined as having serological proof of infection without symptoms of disease. If left untreated, the infection can progress to the symptomatic tertiary stage, with subsequent systemic involvement and potentially serious complications (1, 6). Therefore, early diagnosis is crucial to prevent transmission and to avoid delays in treatment (1).

Although it is typically considered an historic infection, the incidence of syphilis is increasing (7, 8). In 2008, approximately 11 million new cases of infection were reported worldwide (9); from 2005 to 2013, the numbers of primary and secondary syphilis cases per year reported in the United States almost doubled and the annual rate increased from 2.9 to 5.3 cases per 100,000 population (10). In high-income countries, T. pallidum infection rates are specifically increasing among men who have sex with men (11), and high rates of HIV coinfection have also been documented (12, 13). This reemergence warrants renewed attention to the strategies used for the diagnosis and treatment of syphilis (14).

In order to treat syphilis and to prevent its transmission, testing is a common component of prenatal, blood donor, organ donor, and STI screening (3, 4, 15–17). There are many diagnostic tests for syphilis available; however, a commonly accepted standard method is still lacking, and the algorithms used for initial screening and confirmation vary between countries (3, 15–17). T. pallidum cannot be cultured *in vitro*, and direct swabbing and testing of a primary lesion for syphilis are often not possible, as primary lesions are apparent only for a short time and, depending on the location, may go unnoticed by the patient. As a result, the diagnosis of syphilis is usually based on serological methods and typically uses either a treponemal or nontreponemal test for primary screening. The result is subsequently confirmed by retesting, e.g., with an alternative treponemal test and a quantitative nontreponemal test (17). However, this is dependent on the diagnostic algorithm that is being followed. Guidelines currently leave decisions about which tests to use as first-line tests and as tests for confirmation to laboratories and/or clinicians. Historically, the traditional algorithm, which uses a nontreponemal test followed by a treponemal test, has been the standard in many parts of the world. However, the higher sensitivity of recently available enzyme- and chemiluminescence-based immunoassays and the ability to automate such treponemal tests has led to more widespread use of the reverse algorithm (18–21).

Automated treponemal assays such as this are becoming the method of choice in many establishments for the reliable detection of T. pallidum infections among blood donors, facilitating the clear consistent interpretation of results (16). High specificity, especially in potentially cross-reactive samples, is required in order to prevent potential false-positive results, minimizing the need for retesting and reducing patient anxiety. High sensitivity is also required to minimize the likelihood of missing T. pallidum infections at all disease stages. Early detection of infections is extremely important to allow appropriate treatment, as well as the safe and timely supply of blood products. Therefore, a treponemal assay needs to show good seroconversion sensitivity, to reduce the diagnostic window. The availability of multiple automated treponemal tests and their performance data is beneficial for laboratories, supporting broad access to testing and increasing patient and blood safety. This evaluation aimed at further assessing the capabilities of the Elecsys Syphilis assay (Roche Diagnostics, Mannheim, Germany) to fulfill these requirements.

The Elecsys Syphilis assay is a newly developed, fully automated, electrochemiluminescence immunoassay (ECLIA) for the *in vitro* qualitative determination of total antibodies against T. pallidum in human serum and plasma samples (22). The performance of the Elecsys Syphilis assay was previously evaluated with routine clinical samples and blood donations in a multicenter study (22). The aim of the current study was to further assess the performance of the Elecsys Syphilis assay in a broader variety of target populations from Europe and Asia, including those with both low and high (e.g., Spain) rates of reported syphilis cases (23), representative of the diverse environments in which this test will be utilized. The samples included routine screening samples sent by clinical request and blood donation samples, as in the previous study, as well as additional samples from patients with confirmed HIV infections, samples for sexual health screening, and samples from living bone marrow or organ donors. The assay was compared with other routinely used treponemal tests. The study also evaluated the performance of the assay with banked samples, including syphilis-positive and potentially cross-reactive samples, as well as providing the first evaluation of the seroconversion sensitivity of the assay using a commercially available seroconversion panel. The seroconversion sensitivity was further assessed at a specialized center for sexually transmitted infections, with archived serial blood samples from patients with direct diagnoses of primary syphilis, to investigate how early the assay detects serological responses to T. pallidum infections.

## MATERIALS AND METHODS

### Study design.

Seven independent laboratories from different countries (Athens, Greece; Zaragoza, Spain; Milan, Italy; Gävle, Sweden; Porto, Portugal; Kuala Lumpur, Malaysia; and Newcastle, United Kingdom) were involved in the evaluation. The laboratories were chosen to represent a wide variety of different settings, including blood bank testing, routine diagnostic laboratory testing, patient monitoring, general STI screening, prenatal screening, sexual health screening, and transplantation screening. Not all investigations were performed by every center. All samples were deidentified or coded prior to use in this study.

Samples for the assessment of specificity were fresh serum or plasma samples left over from blood donations or routine diagnostic requests, including samples for the diagnosis of patients with suspected syphilis, prenatal screening, general STI screening, sexual health care screening, and treatment monitoring. At most centers, samples were unselected; however, only samples from first-time blood donors were included among the samples from the blood and transplantation center in Porto, Portugal.

Sensitivity was assessed at the centers in Athens, Gävle, Newcastle, Porto, and Zaragoza, using enriched cohorts of archived samples that had been confirmed previously as syphilis positive with two treponemal tests (see Table S1 in the supplemental material). For confirmed-positive samples with a defined clinical stage (Newcastle), the stage was determined by the clinician who provided the samples for testing.

The specificity and/or sensitivity of the assay was also assessed with special cohorts, including samples from living organ and bone marrow donors, confirmed HIV-positive samples, and selected archived potentially cross-reacting samples that had been found previously to be false positive with comparator assays. The potentially cross-reacting samples originated from blood donations and were characterized by syphilis test results only. Further information regarding the type of samples assessed and the comparator and confirmatory assessments performed at each specific center are presented in Table S1 in the supplemental material.

### Elecsys Syphilis assay.

The Elecsys Syphilis assay, based on electrochemiluminescence technology, simultaneously detects antitreponemal IgG and IgM antibodies. The samples are incubated with a mixture of biotinylated and ruthenylated thermostable recombinant TpN15, TpN17, and TpN47 antigens. The presence of the corresponding antibodies subsequently leads to the formation of double-antigen sandwich (DAGS) immune complexes. The addition of streptavidin-coated paramagnetic microparticles causes the immune complexes to bind to the solid phase due to biotin-streptavidin interactions. The microparticles are then magnetically captured on the electrode surface, and any unbound material is removed. Chemiluminescence is then induced by voltage application and measured with a photomultiplier. The total assay time is 18 min, and the results are calculated automatically by the analyzer software.

### Comparator assays.

Each center evaluated the Elecsys Syphilis assay and at least one of the following treponemal comparator assays: Architect Syphilis TP assay (Abbott Laboratories, Wiesbaden, Germany), Liaison Treponema Screen assay (DiaSorin, Saluggia, Italy), Serodia T. pallidum particle agglutination (TPPA) assay (Fujirebio, Tokyo, Japan), and Mediace T. pallidum latex agglutination (TPLA) assay (Sekisui Medical, Tokyo, Japan). All comparator assays were performed according to the manufacturers' instructions. In Milan, the Architect Syphilis TP assay was used to initially characterize samples that were later retested with the Elecsys Syphilis assay; therefore, this was not a simultaneous comparator assay.

### Methods and analyses.

The Elecsys Syphilis ECLIA results were expressed as signal/cutoff (s/co) ratios, with s/co ratios of <1.0 indicating nonreactive results and s/co ratios of ≥1.0 reactive results. Samples with initial nonreactive results were considered negative for T. pallidum antibodies. According to the manufacturer's instructions, all samples with initial reactive results were retested in duplicate using the Elecsys Syphilis assay and were considered to be (repeatedly) reactive if either of the results demonstrated an s/co ratio of ≥1.0. The samples were then subjected to confirmatory testing.

For the majority of comparator assays, the results were also expressed as s/co ratios and were interpreted according to the manufacturers' guidelines. The exceptions were the Mediace TPLA assay and Serodia TPPA assay, which are agglutination-based assays. In the case of the Mediace TPLA assay, the results were expressed as titer units; for the Serodia TPPA assay, the agglutination patterns were inspected and the results expressed as titers. Results for both assays were interpreted according to the manufacturers' guidelines. Samples that were initially reactive or equivocal using one of the comparator assays were retested either in duplicate or singly, as specified in the information for users or according to the established laboratory routines in the blood banks (retesting in duplicate). Samples were considered to be (repeatedly) reactive if at least one of the repeat tests yielded reactive results, and such samples were subjected to confirmatory testing. The routine testing laboratories (Athens and Milan) followed the instructions for users for the Architect Syphilis TP assay (no retesting of reactive samples).

This study used a wide assortment of confirmatory tests, locally available to each center (see Table S1 in the supplemental material). Samples were classified as negative when all available test results were nonreactive and positive when all treponemal test results were reactive. A sample was considered indeterminate when the confirmatory tests showed discrepant results and no consensus could be reached. Samples with indeterminate confirmation results were excluded from the evaluations of sensitivity and specificity.

### Assessment of seroconversion sensitivity.

SeraCare syphilis seroconversion panel PSS901 is a 9-member panel of undiluted, naturally occurring plasma samples, collected from a 41-year-old woman in the United States over 60 days in 2012. The samples convert from negative to positive results for both nontreponemal and treponemal tests, demonstrating an early T. pallidum infection. This panel was assessed using the Elecsys Syphilis assay at the centers in Gävle, Porto, Milan, and Newcastle. The seroconversion panel was also assessed with the relevant comparator test used at the centers in Gävle, Porto, and Newcastle. For comparison with additional available assays, the data used were as reported in the seroconversion panel package information.

At the center in Milan, archived serial blood samples collected from patients with direct diagnoses of primary syphilis, confirmed using dark-field microscopy and prior or past seroconversion based on the routine serological testing in the laboratory, were tested with the Elecsys Syphilis assay. These samples were collected at the time of diagnosis and as available during treatment monitoring.

## RESULTS

### Numbers of samples tested.

A total of 2,006 samples from routine diagnostic requests, 5,811 blood donation samples, 34 samples from bone marrow/organ donors, and 103 archived potentially cross-reacting samples were assessed for specificity. A total of 41 routine clinical samples and 8 blood donor samples were found to be reactive using the Elecsys Syphilis assay and comparator assays and were subsequently confirmed to be syphilis positive (Tables 1 and 2). No routine clinical samples were found to be indeterminate. However, 11 blood donor samples that were found to be reactive with the Architect and/or Elecsys Syphilis assays showed indeterminate confirmation results. Samples with positive or indeterminate confirmation results were disregarded, and specificity was assessed with the remaining subset of the cohorts (total negative samples in Tables 1 and 2). Confirmed-positive samples were included in the assessments of sensitivity at the respective centers, if appropriate.

**TABLE 1:** Specificity of Elecsys Syphilis assay and comparator assays with routine clinical samples, by individual study center

Testing site and parameter^*a*^	Elecsys Syphilis	Architect Syphilis TP	Liaison Treponema Screen
Newcastle, United Kingdom			
No. of samples			
Total tested	1,006		1,006
Confirmed positive	32		32
Indeterminate	0		0
Total negative	974		974
False positive	2		1
Specificity (95% CI) (%)	99.79 (99.26–99.98)		99.90 (99.43–100.00)
Athens, Greece			
No. of samples			
Total tested	1,000	1,000	
Confirmed positive	9	9	
Indeterminate	0	0	
Total negative	991	991	
False positive	1	1	
Specificity (95% CI) (%)	99.90 (99.44–100.00)	99.90 (99.44–100.00)	
Overall			
No. of samples			
Total tested	2,006	1,000	1,006
Confirmed positive	41	9	32
Indeterminate	0	0	0
Total negative	1,965	991	974
False positive	3	1	1
Specificity (95% CI) (%)	99.85 (99.55–99.97)	99.90 (99.44–100.00)	99.90 (99.43–100.00)

a Confirmed positive samples were excluded from the specificity assessment. False-positive samples represent a subset of the total negative samples. Specificity was calculated as the percentage of negative samples correctly identified as nonreactive, i.e., (total no. negative − no. false positive)/total no. negative. CI, confidence interval (2 sided); TPLA, Treponema pallidum latex agglutination; TPPA, Treponema pallidum particle agglutination.

**TABLE 2:** Specificity of Elecsys Syphilis assay and comparator assays with blood donation samples, by individual study center

Testing site and parameter^*a*^	Elecsys Syphilis	Architect Syphilis TP	Mediace TPLA	Serodia TPPA
Gävle, Sweden				
No. of samples				
Total tested	1,021	1,021	1,021	
Confirmed positive	0	0	0	
Indeterminate	1	1	1	
Total negative	1,020	1,020	1,020	
False positive	0	2	4	
Specificity (95% CI) (%)	100.00 (99.64–100.00)	99.80 (99.29–99.98)	99.61 (99.00–99.89)	
Kuala Lumpur, Malaysia				
No. of samples				
Total tested	1,112			1,112
Confirmed positive	3			3
Indeterminate	0			0
Total negative	1,109			1,109
False positive	1			1
Specificity (95% CI) (%)	99.91 (99.50–100.00)			99.91 (99.50–100.00)
Porto, Portugal				
No. of samples				
Total tested	2,099	2,099		
Confirmed positive	5	5		
Indeterminate	8	8		
Total negative	2,086	2,086		
False positive	0	5		
Specificity (95% CI) (%)	100.00 (99.82–100.00)	99.76 (99.44–99.92)		
Zaragoza, Spain				
No. of samples				
Total tested	1,579	1,579		
Confirmed positive	0	0		
Indeterminate	2	2		
Total negative	1,577	1,577		
False positive	7	3		
Specificity (95% CI) (%)	99.56 (99.09–99.82)	99.81 (99.45–99.96)		
Overall				
No. of samples				
Total tested	5,811	4,699	1,021	1,112
Confirmed positive	8	5	0	3
Indeterminate	11	11	1	0
Total negative	5,792	4,683	1,020	1,109
False-positive	8	10	4	1
Specificity (95% CI) (%)	99.86 (99.73–99.94)	99.79 (99.61–99.90)	99.61 (99.00–99.89)	99.91 (99.50–100.00)

a Confirmed-positive and indeterminate samples were excluded from the specificity assessment. False-positive samples represent a subset of the total negative samples. Specificity was calculated as the percentage of negative samples correctly identified as nonreactive, i.e., (total no. negative − no. false positive)/total no. negative. CI, confidence interval (2 sided); TPLA, Treponema pallidum latex agglutination; TPPA, Treponema pallidum particle agglutination.

Sensitivity was assessed with 333 confirmed-positive samples and 23 samples from patients with primary syphilis. Two samples from the center in Athens were excluded from the sensitivity assessment due to negative confirmation results after they had initially been found to be reactive with the Architect Syphilis TP assay. Sixty-nine samples from patients with confirmed HIV infections were assessed for Elecsys Syphilis assay specificity and sensitivity.

### Specificity.

The overall specificity of the Elecsys Syphilis assay with 1,965 routine clinical samples was 99.85% (Table 1), and that with 5,792 blood donor samples was 99.86% (Table 2). The performance of the Elecsys Syphilis assay at each individual testing center is also presented (Tables 1 and 2). Among 34 patients considered for bone marrow transplantation or organ donation, the specificity of the Elecsys Syphilis assay was 97.06%. Among 52 syphilis-negative patients with confirmed HIV infections, the specificity of the Elecsys Syphilis assay was 100% (Table 3). The specificity of the Elecsys Syphilis assay with 103 potentially cross-reacting samples was 100% (2-sided 95% confidence interval [CI], 96.84 to 100.00%). These samples were all found previously to be false-positive samples using comparator assays.

**TABLE 3:** Performance of Elecsys Syphilis assay with special cohorts

Patient group and parameter^*a*^	Elecsys Syphilis	Liaison Treponema Screen
Bone marrow/organ donors		
No. of samples		
Total tested	34	34
Total negative	34	34
False positive	1^*b*^	0
Specificity (95% CI) (%)	97.06 (84.67–99.93)	100.00 (89.72–100.00)
Patients with confirmed HIV infections		
No. of samples		
Total tested	69	69
Confirmed positive	17	17
Total negative	52	52
False positive	0	0
False negative	0	0
Specificity (95% CI) (%)	100.00 (93.15–100.00)	100.00 (93.15–100.00)
Sensitivity (95% CI) (%)	100.00 (80.94–100.00)	100.00 (80.94–100.00)

a Specificity was calculated as the percentage of negative samples correctly identified as nonreactive, i.e., (total no. negative − no. false positive)/total no. negative. Sensitivity was calculated as the percentage of positive samples correctly identified as reactive, i.e., (no. confirmed positive − no. false negative)/no. confirmed positive. CI, confidence interval (2 sided).

b One sample was found to be reactive with the Elecsys Syphilis assay and a second tube from the same blood draw was weakly positive with the Elecsys Syphilis assay; therefore, the donor was considered to be repeatedly reactive with the Elecsys Syphilis assay.

### Sensitivity.

A total of 267 confirmed-positive routine diagnostic samples and 64 confirmed-positive blood donor samples tested reactive with the Elecsys Syphilis assay (s/co ratios ranging from 1.27 to 390.5 [average, 121.7; median, 112.1]). The sensitivity of the Elecsys Syphilis assay was 100% (2-sided 95% CI, 98.89 to 100.00%). Confirmed-positive samples from the Freeman Hospital in Newcastle were categorized by disease stage, with 39 primary stage, 44 secondary stage, 44 latent period, and 2 late latent period samples. The sensitivity of the Elecsys Syphilis assay was 100% during each stage of the disease. The sensitivity of the Elecsys Syphilis assay in samples from 17 syphilis-positive patients with confirmed HIV infections was 100% (Table 3).

### Seroconversion sensitivity.

**FIG 1:**
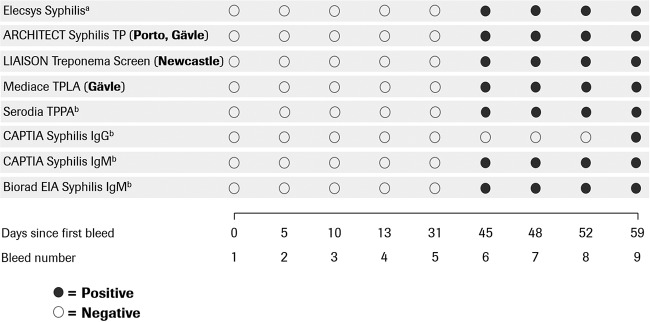
Assessment with SeraCare syphilis seroconversion panel PSS901. Day 0 represents the time at which the first blood sample was collected, and all subsequent samples are referred to by the number of days after the first bleed at which they were collected. ^a^ Tested in Porto, Newcastle, Gävle, and Milan. ^b^ These data are from the seroconversion panel package insert. The comparator assays used and the centers performing the assessment are indicated. EIA, enzyme immunoassay; TPLA, Treponema pallidum latex agglutination; TPPA, Treponema pallidum particle agglutination.

In the assessment of seroconversion sensitivity with SeraCare seroconversion panel PSS901, the Elecsys Syphilis assay detected T. pallidum infection in the same bleed as all comparator assays, with the exception of the Captia Syphilis IgG assay (Trinity Biotech, Jamestown, NJ), which detected infection 14 days later (Fig. 1). The seroconversion sensitivity of the Elecsys Syphilis assay was also assessed with 23 samples collected at different intervals from 14 patients with primary syphilis confirmed via dark-field microscopy (Table 4). Among the samples collected on the day of diagnosis, 10 patients were found to be reactive for T. pallidum antibodies with both the Elecsys Syphilis assay and the Architect Syphilis TP assay, while one patient was found to be nonreactive with both assays. Three patients were found to be reactive with the Elecsys Syphilis assay although they had been found to be nonreactive with the Architect Syphilis TP assay, albeit with slightly elevated signals (below the cutoff value). For one of those patients, an additional sample collected 4 weeks later was subsequently found to be reactive with both the Architect and Elecsys assays. The s/co ratios for reactive samples from the Elecsys Syphilis assay ranged from 7.83 to 187.8 (average, 44.7; median, 28.7).

**TABLE 4:** Performance of Elecsys Syphilis assay and comparator assay with samples from 14 patients diagnosed with primary syphilis via dark-field microscopy^*a*^

Elecsys Syphilis assay result	No. with Architect Syphilis TP assay result of:	
	Reactive	Negative
Reactive	10	3
Negative	0	1

a The samples were collected on the day of diagnosis.

## DISCUSSION

This analysis confirmed the high specificity and sensitivity of the Elecsys Syphilis assay for assessment of routine diagnostic samples and blood donor samples. This analysis also established the performance of the assay in a wide range of populations, including populations in settings with low and high rates of syphilis, and with a variety of different samples, including samples from sexual health and transplantation screening environments. The study also provided the first evaluation of the seroconversion sensitivity of the Elecsys Syphilis assay. During this study, an extremely broad range of confirmatory assays were used at each center to gain the most information for accurate classification of samples, in order to avoid introducing bias into the assessment and to provide an accurate analysis of the performance of the screening test.

For samples from blood donors and routine diagnostic requests, the findings presented in this study agree with those of a previous performance evaluation of the Elecsys Syphilis assay, in which the specificity and sensitivity were found to be 99.88% with 8,063 negative samples from target cohorts, i.e., blood donation and routine testing samples, and 99.57 to 100.00% with 928 banked syphilis-positive samples, respectively (22). The specificity and sensitivity of the comparator assays evaluated in this study are also in agreement with previously published studies (22, 24–28).

In the blood donor setting at the centers in Gävle and Porto, the Elecsys Syphilis assay displayed high specificity. Along with high sensitivity, high specificity is of great importance in a blood screening environment, where any donations with nonspecific serological screening results that cannot be confirmed with a supplementary assay are usually eliminated. The results from this study also indicated that the specificity of the Elecsys Syphilis assay was 100% with potentially cross-reacting blood donation samples that were found previously to be false positive with comparator assays, which highlights the good performance of this assay. Low rates of biological false-positive results will help prevent unnecessary disposal of healthy blood. There was a larger number of false-positive samples with the Elecsys Syphilis assay at the center in Zaragoza, compared with the other centers, although with a reasonable overlap of the CIs with the previous performance evaluation (95% CI, 99.77 to 99.94% [22]). In order to explain fully this variation in performance and to provide clarification, further studies in Spain are recommended.

The specificity of the Elecsys Syphilis assay was 97.06% with bone marrow/organ donor samples, due to one false-positive result. The investigator who assessed that sample found that the donor was considered at high risk due to lifestyle choices and provided indeterminate results for other predonation testing. Limited conclusions can be drawn about the specificity of the Elecsys Syphilis assay in this cohort, however, due to the small number of samples available (*n* = 34). Further studies would be recommended in order to investigate the specificity of the Elecsys Syphilis assay in this population.

HIV can adversely affect the serological responses to syphilis and may affect the diagnosis of T. pallidum infections (29). Previous studies showed the reliable detection of syphilis using the Elecsys Syphilis assay among patients coinfected with HIV (22). The data presented in this study confirm those results, although it should again be noted that the sample size was relatively small (*n* = 69). The high specificity and sensitivity of the Elecsys Syphilis assay among patients with HIV infections are extremely important, particularly because syphilis coinfections among patients with HIV are not uncommon (30–32) and data suggest that infection with syphilis may, in fact, increase the susceptibility to HIV infection (30).

The Elecsys Syphilis assay showed high sensitivity with confirmed-positive samples at all stages of disease, including the early and latent stages, during which titers of antibodies against T. pallidum can be low and hard to detect. Early detection of syphilis is vital to prevent further transmission of the infection and to allow appropriate, effective, and timely treatment. The TpN47 antigen was specifically included in this assay to capture early IgM and to increase early seroconversion sensitivity, as TpN47 is highly immunogenic and activates the early immune response (33).

To our knowledge, this study is the first evaluation of the seroconversion sensitivity of the Elecsys Syphilis assay. SeraCare recently released a seroconversion panel for syphilis, allowing comparison using a variety of different syphilis assays. With this panel, all treponemal tests (with the exception of the Captia Syphilis IgG assay) detected syphilis in the same bleed. However, there was a period of 14 days between the first reactive bleed in the panel and the previous bleed for which a sample was available. Therefore, the panel does not cover the window of seroconversion very effectively. If bleeds at interim time points were available, then differences in seroconversion sensitivity might have become apparent.

Moreover, the data from the Milan center indicated a potential trend for earlier detection of infection in 3 of 14 patients in early seroconversion by using the Elecsys Syphilis assay versus the Architect Syphilis TP assay (Table 4). One patient was found to be nonreactive with both assays, although routine testing with the Treponema pallidum hemagglutination (TPHA) assay and rapid plasma reagin (RPR) test yielded positive results. Therefore, this patient was considered to be in the period of seroconversion, with incomplete seropositivity. Given the relatively small sample size, further investigation is required before robust conclusions can be drawn.

Serological tests are the foundation of syphilis management, and they must meet strict standards before they can be incorporated into routine screening programs (19). High sensitivity is extremely important to prevent false-negative results and to ensure correct identification of infected individuals. High specificity is also extremely important for diagnostic assays, in order to minimize false-positive results and unnecessary confirmatory testing that may be costly (34) or lead to indeterminate results that may present clinicians with difficult treatment decisions.

This study confirms the high specificity and sensitivity of the Elecsys Syphilis assay with a variety of clinical samples from populations in both routine diagnostic laboratory and blood bank settings. The data presented provide further evidence for the use of treponemal tests as first-line screening tools for the detection of syphilis, facilitating the correct detection of T. pallidum infections and minimizing the rates of biological false-positive results.

## Supplementary Material

Supplemental material
