# Supplementary material for: Evaluation of Elecsys Syphilis Assay for Routine and Blood Screening and Detection of Early Infection
Source: J Clin Microbiol. 2016 Aug 24;54(9):2330–6. doi: 10.1128/JCM.02544-15 (PMC5005501; doi:10.1128/JCM.02544-15)
Supplement: Supplemental material [file supp_54_9_2330__index.html]

Supplemental material 

# Evaluation of Elecsys Syphilis Assay for Routine and Blood Screening and Detection of Early Infection

## Supplemental material

- Supplemental file 1 -

  Table S1 (Comparator assays, confirmation methods, and samples tested at each of the centers)

  PDF, 41K
