# Supplementary material for: Evaluation of Elecsys Syphilis Assay for Routine and Blood Screening and Detection of Early Infection
Source: J Clin Microbiol. 2016 Aug 24;54(9):2330–6. doi: 10.1128/JCM.02544-15 (PMC5005501; doi:10.1128/JCM.02544-15)
Supplement: Supplemental material [file JCM.02544-15_zjm999095133so1.pdf]

TABLE S1 Comparator assays, confirmation methods and samples tested at each of the centers

**Center**	**Comparator assay**	**Confirmation method(s)**	**Samples**
Athens, Greece	ARCHITECT Syphilis TP (Abbott)	FTA-Abs total + IgM (BioMerieux); TPHA (Siemens); Immunoblot IgG/IgM (Mikrogen); RPR (Axis Shield)	1,000 fresh serum samples from routine diagnostic requests (prenatal and general STI screening, diagnosis of patients and treatment monitoring);<br>131 archived confirmed-positive serum samples (based on FTA-Abs total + IgM and/or TPHA)
Newcastle, UK	LIAISON Treponema Screen (DiaSorin; routinely used at this center)	Serodia TPPA (Fujirebio); RPR (Lab21); EIA IgM (Lab21); Immunoblot IgG/IgM (Mikrogen)	1,006 fresh serum samples from routine diagnostic requests (sexual healthcare screening);<br>69 fresh serum samples with confirmed HIV infection;<br>34 fresh serum samples from organ and bone marrow donors;<br>97 archived confirmed-positive serum samples (based on LIAISON Treponema Screen and TPPA; defined clinical stage: primary, secondary, latent, late latent)

Zaragoza, Spain	ARCHITECT Syphilis TP (Abbott; routinely used at this center)	FTA-Abs IgG/IgM (Euroimmun); TPHA (Randox); VDRL (Siemens); Immunoblot IgG/IgM (Mikrogen)[a,b]	1,579 unselected fresh blood donation serum samples (~10% first-time donors);<br>88 archived potentially cross-reactive plasma samples from blood donations (corresponding serum sample: false-positive ARCHITECT Syphilis TP and negative FTA-Abs IgG/IgM);<br>32 archived confirmed-positive plasma samples from blood donations (corresponding serum sample: positive ARCHITECT Syphilis TP and FTA-Abs IgG/IgM)
Gävle, Sweden	ARCHITECT Syphilis TP (Abbott); Mediace TPLA (Sekisui; routinely used at this center)	FTA-Abs IgG/IgM (BioMerieux)[c]; Serodia TPPA (Fujirebio); TPHA (Siemens)[c]; RPR (BD); Immunoblot IgG/IgM (Mikrogen)[c]	1,021 unselected fresh blood donation serum samples (~5% first-time donors);<br>15 archived potentially cross-reactive serum samples from blood donations (false-positive Mediace TPLA and negative TPPA);

			2 archived confirmed-positive serum samples (based on Mediace TPLA and TPPA)
Porto, Portugal	ARCHITECT Syphilis TP (Abbott; routinely used at this center)	TPHA (Bio-Rad); ELISA IgG/IgM (Diesse); Immunoblot IgG/IgM (Mikrogen)[d]	2,099 fresh blood donation serum samples (first-time donors);<br>25 archived confirmed-positive serum samples (based on ARCHITECT Syphilis TP and ELISA IgG)
Kuala Lumpur, Malaysia	Serodia TPPA (Fujirebio)	RPR (Macro-Vue); Immunoblot IgG/IgM (Mikrogen)[c]; FTA-Abs IgG/IgM (BioMerieux)[c]	1,112 unselected fresh blood donation plasma samples (~50% first-time donors)
Milan, Italy	ARCHITECT Syphilis TP (Abbott; routinely used at this center. Samples were pre-characterized using this method – no assessments with this		23 archived serum samples from 14 patients with primary syphilis to assess seroconversion sensitivity (positive darkfield microscopy from primary lesion)

	assay were performed<br>during the study)

EIA, enzyme immunoassay; ELISA, enzyme-linked immunosorbent assay; FTA, fluorescent treponemal antibody; RPR, rapid plasma reagin; STI, sexually transmitted infection; TPHA, *Treponema pallidum* hemagglutination assay; TPPA, *Treponema pallidum* particle agglutination; VDRL, venereal disease research laboratory

All archived, frozen samples were only thawed once prior to testing

[a] These assays were performed by the Reference Laboratory, Barcelona, Spain

[b] The immunoblot IgG/IgM (Mikrogen) for 8 samples from Zaragoza was additionally performed by Roche Diagnostics, Penzberg, Germany

[c] These assays were performed by the Biomedicine SA laboratory, Athens, Greece

[d] One sample from Porto was additionally tested with immunoblot IgG/IgM (Mikrogen), FTA-Abs total + IgM (BioMerieux) and RPR (Axis Shield) by Roche Diagnostics, Penzberg, Germany and the Biomedicine laboratory, Athens, Greece
